# 

**Published:** 1878-07

**Authors:** 


### TABLE OF CONTENTS.

### ORIGINAL COMMUNICATIONS.

UTERINE TROUBLE AND ITS TREATMENT. *By a Country M. D.* ........ 193
A PECULIAR DEFORMITY. *By H. A. Kiker, M. D., Tallapoosa, Ga.* .. 106
TYPHOID FEVER. *By J. R. Groover, M. D., Mica, Ga.* ............... 197

### REPORTS OF SOCIETIES.

Baltimore Academy of Medicine ............................ 197
Medical and Surgical Society of Baltimore ........................ 206
Proceedings of the Gynæcological Society of Boston ............... 212

### SELECTIONS.

Miner's Wrist ............................................ 213
Case of Intestinal Obstruction by a Diverticulum Verum of Meckel. 215
Rheumatoid Inflammation of the Joints in Women ................ 217
Distinctive Lists of an Insane and Sane Delusion ................ 223
Gastric Ulcer ............................................ 225
Hints on the Hypodermic Use of Morphia ........................ 228
Jaborandi and its Active Principle Pilocarpine ................... 231

### EDITORIAL.

American Medical Association .............................. 234
Medical College Association ................................ 241
European Correspondence ................................... 245
Editorial Correspondence .................................. 250
Justice Rendered ......................................... 253
Medical Association of Georgia ............................. 253

### EXCERPTA.

Carbolic Acid Spray in Coughs .............................. 254
Can Syphilis be Transmitted by means of the Spermatic Fluid? ... 255
Medical uses of the Telephone ............................. 255
Membranous Dysmenorrhœa ................................. 256

